# Association of* ACE I/D* and *AGTR1 A1166C* Gene Polymorphisms and Risk of Uterine Leiomyoma: A Case-Control Study

**DOI:** 10.31557/APJCP.2019.20.9.2595

**Published:** 2019

**Authors:** Farshid Keshavarzi, Batool Teimoori, Farahnaz Farzaneh, Mojgan Mokhtari, Darya Najafi, Saeedeh Salimi

**Affiliations:** 1 *Department of Clinical Biochemistry,*; 3 *Department of Obstetrics and Gynecology, School of Medicine,*; 2 *Cellular and Molecular Research Center,*; 4 *Pregnancy Health Research Center, Zahedan University of Medical Sciences, Zahedan, *; 5 *Department of Obstetrics and Gynecology, School of Medicine,*; 6 *Medical College, Iran University of Medical Sciences, Tehran, Iran. *

**Keywords:** ACE, AGTR1, polymorphism, uterine leiomyoma

## Abstract

**Objective:**

Uterine leiomyoma (UL) can be considered as the most common benign gynecological tumors of the smooth muscle cells in the myometrium. They are likely to be associated with infertility and recurrent abortion as well as obstructed labor and post-partum hemorrhage. Moreover, altered vascular-related genes can be linked to developing leiomyoma. Polymorphisms of the angiotensin-converting enzyme (ACE) gene are associated with some vascular diseases. The present study was carried out to investigate the association of *ACE I/D* and *AGTR1* *A1166C* gene polymorphisms and the risk of uterine leiomyoma in a sample of Iranian population.

**Methods:**

The study was carried out on a total of 413 women divided into 202 patients with diagnosed uterine leiomyomas and a control group of 211. Genotyping was performed using the PCR or PCR-RFLP methods.

**Results:**

The ID and DD genotypes of *ACE I/D* polymorphism were associated with 2 and 2.9 fold higher risk of UL compared to II genotype (OR, 2 [95% CI, 1.3 to 3.2]; P = 0.004 and OR, 2.9 [95% CI, 1.6 to 5]; P = 0.0002). The frequencies of *ACE* D alleles were 53.7% in women with UL and 40.3% in controls, which were observed to be statistically different (P < 0.0001). The alleles and genotypes of* AGTR1 A1166C* polymorphism were not different between UL and control women (P=0.9).

**Conclusion:**

The *ACE *ID and DD genotypes were associated with a higher risk of UL. No relationship was found between *AGTR1* *A1166C* polymorphism and UL.

## Introduction

Uterine leiomyomas (ULs) can be considered as the most common benign monoclonal tumors of the smooth muscle cells in the myometrium (Flynn et al., 2006). Evidence suggests that 70% of women may develop uterine fibroids. Although this disorder may be without signs and symptoms, in 40 to 50 percent of women over age 35 it may present as menorrhagia, infertility, pain, and recurrent pregnancy loss (RPL) (Marino et al., 2004; Wang et al., 2015). There are different risk factors influencing the growth of UL, including: ethnicity, smoking, family history, obesity, diet rich in meat, oral contraceptive pills, age, and biological biomarkers (Faerstein et al., 2001; Keshavarzi et al., 2017). Despite the various studies conducted to understand UL etiology, the exact mechanism of UL pathogenesis is not yet known clearly (Strawn et al., 1995). Several mechanisms have been suggested that have the effects on growth of UL, including ovarian angiogenesis, steroid hormones, growth factors, and apoptosis related factors (Wang et al., 2002).

Both abnormal angiogenesis and vascular-related growth factors have been considered to be associated with the UL pathogenesis and growth. Growth factors may typically stimulate the angiogenesis of leiomyoma cells as compared with adjacent normal cells (Boehm et al., 1990; Di Lieto et al., 2005). Numerous investigations have revealed that both chromosome abnormalities in UL patients as well as genetic factors play key roles in UL pathogenesis in different countries, such as Iran (Gan et al., 2015; Salimi et al., 2015; Yaghmaei et al., 2015; Salimi et al., 2016). It is believed that ACE activity may be related to tumor growth and ACE inhibitors as well as angiotensin receptor blockers, thereby contributing to the suppression of tumor growth.

The renin-angiotensin system (RAS) may be considered as an essential pathway in the regulation of blood pressure and electrolyte balance. Independent tissue renin angiotensin systems (RASs) have been demonstrated in many organs including heart, kidney, brain, adrenal glands, vasculature, and the uteroplacental unit (Kobori et al., 2007). In this pathway, the angiotensin peptide binds to its related angiotensin receptors, angiotensin receptor type 1 (AGTR1) and angiotensin receptor type 2 (AGTR2) to prompt various biological responses (Elton et al., 2010). Although several genes are selectively overexpressed in leiomyomas, compared to normal myometrium, such as insulin-like growth factor-2 receptor and insulin-like growth factor binding protein, the angiotensinogen gene as a member of renin-angiotensin system has been down regulated in these tissues. There is evidence showing that that both ACE inhibition and AGTR1 blockade inhibit tumor angiogenesis, vascular density, tumor growth, reduced tumor volume, cell proliferation, and mitotic index and they actually reduce metastasis, too.

Since the effects of polymorphisms may be reversed or antagonized using medical treatment, they can be used to reduce or to inhibit tumor development (Hortobagyi, 2012). It has been shown that AGTR1 protein is expressed in benign states, such as ovarian cyst adenomas, and is involved in angiogenesis and tumor progression. It is also reported to be expressed in several cancers, including the breast (Herr et al., 2008), bladder (Kosugi et al., 2006), gastric (Röcken et al., 2007), pancreatic (Amaya et al., 2004), prostate (Uemura et al., 2006), endometrial (Watanabe et al., 2003), and renal cancers as well as ovarian carcinoma (Suganuma et al., 2005). Angiotensin type 1 receptor is mostly up regulated during the progression from normal to malignant phenotypes, indicating a relationship between the RAS and tumor progression at the very least. The angiotensin converting enzyme (ACE; EC 3.4.15.1), a dipeptidyl carboxy peptidase, is encoded by the *ACE* gene, which is located on chromosome 17q23 and includes 25 introns and 26 exons (Sayed-Tabatabaei et al., 2006a). ACE enzyme, which catalases conversion of the inactive angiotensin I to the angiotensin II, exerts most of its effects via the activation of AGTR1 receptors expressed in vascular smooth muscle cells and adrenal glands, among others (Irani and Xia, 2008).

There is an insertion/deletion polymorphism (*I/D*) of a 287 bp in the intron 16 of *ACE* gene, with its DD genotype, which may be associated with elevated plasma and serum ACE levels as compared to heterozygous ID and homozygous II genotypes. In addition, the A to C polymorphism in the 3ˊ-’untranslated region at nucleotide 1,166 of the *AGTR1* gene has been identified and described in association with various diseases.

As the role of RAS system has been characterized in tumor development, the aim of the present study was to investigate the association between the* ACE I/D*, and* A1166C* polymorphisms and UL.

## Materials and Methods

*Subjects*

A total of 413 pre-menopausal women including 202 uterine leiomyoma and 211 healthy controls were recruited in the current case-control study. The case and control groups were matched according to age, ethnicity, and BMI. Participants were selected from among women who had undergone myomectomy or hysterectomy and were confirmed pathologically in Ali-ebn-Abitaleb Hospital. The controls selected from among women referring for routine check-ups who had no history of UL upon sonography or examination. UL women and controls had no history of malignancy and systemic diseases.

Each research participant voluntarily provided her informed consent with her peripheral blood sample. The project protocols followed the principles stated in the Declaration of Helsinki for medical research involving human subjects and received its prior approval from the Ethics Committee of Zahedan University of Medical Sciences (Code no. 8807). 

*Genotype analysis*

Genomic DNA was extracted from 2 mL of peripheral blood leucocytes from all women for genetic analysis using the salting out method. In the current study, two polymorphic sites were analyzed in both uterine leiomyoma and the control groups. 

*Genotyping for I/D polymorphism of ACE gene*

To determine the *ACE I/D* gene polymorphism, a genomic DNA fragments on intron 16 of the ACE gene was amplified using PCR. Two oligonucleotide primers (forward) 5ˊ-CTG GAG AGC CAC TCC CAT CCT TTC T-3ˊ and (reverse) 5ˊ- GGG ACG TGG CCA TCA CAT TCG TCA G-3ˊ were used in a 20-μL final volume for each amplification. Each PCR reaction consisted of an initial cycle at 94°C for 5 min, 30 cycles at 94°C for 30 s, 60°C for 60 s, and 72°C for 60 s; followed by an extension at 72°C for 10 min. The PCR products of two alleles of 490 bp and 190 bp were electrophoresed in 2% agarose gels, and visualized under ultraviolet (UV) light using ethidium bromide staining. A 190 bp fragment was produced in the absence of an insertion (D) and a 490 bp fragment in the presence of insertion (I). Thus, the homozygote DD produced one band (190 bp), the homozygote II produced one band (480 bp long), and the heterozygote ID produced both bands (190 bp and 490 bp). The feedback yielded a 335-bp amplicon only in the existence of an I allele and no product in homozygous for DD.

**Table 1:** Clinical and Demographic Characteristics of UL Women and Control Group

	UL women (n=202)	Controls (n=211)	P -value
Maternal age (years)	38.8±9.9	38.3±7.8	NS
Marriage status, n (%)	188 (93)	203 (96)	NS
BMI (Kg/m^2^)	25.9±5.5	25.1±4.4	NS
Age of menarche (years)	13.6± 1.7	13.2±1.4	NS
Duration of menses (days)	6.2±1.7	5.8±1.6	NS
Menstrual cycle (days)	28.3± 3.5	28.5±2.8	NS
Bleeding, n (%)	121 (60)	7 (3)	<0.0001
Pain, n (%)	58 (29)	13 (6)	<0.0001

NS, (not significant); UL, (uterine leiomyoma)

**Table 2:** The Allelic and Genotypic and Frequencies of *ACE I/D* and *Angiotensin II Type-1 Receptor A1166C *Polymorphisms in UL Women and Controls

Genotypes/Alleles	Uterine Leiomyoma (n=202)	Control (n=211)	P-value	OR (95% CI)
*ACE I/D*				
II	42 (20.8)	78 (37)		1
ID	103 (51)	96 (45.5)	0.004	2 (1.3 –3.2)
DD	57 (28.2)	37 (17.5)	0.0002	2.9 (1.6-5)
I	187 (46.3 )	252 (59.7)	-	-
D	217 (53.7)	170 (40.3)	<0.0001	1.7 (1.3-2.3)
*AGTR1 A1166C*				
AA	175 (86.6)	182 (86.2)		
AC	27 (13.4)	28 (13.3)	1	1 (0.6-1.8)
CC	0 (0)	1 (0.5)	-	-
A	377 (93.3)	392 (92.9)		
C	27 (6.7)	30 (7.1)	0.9	0.9 (0.6-1.6)

*Genotyping for the A1166C polymorphism of AGTR1 gene*

This polymorphic site was genotyped using polymerase chain reaction-restriction fragment length polymorphism (PCR-RFLP) method. The primers used to amplify DNA fragment, encompassing the A1166C polymorphism included 5ˊ-AAT GCT TGT AGC CAA AGT CAC CT-3ˊ and (reverse) 5ˊ-GGC TTT GCT TTG TCT TGT TG-3ˊ to produce a fragment of 856 bp. PCR was performed in a 20 μl final volume. After an initial melting step at 94°C for 5 min, the PCR procedure (consisting of 35 steps) was carried out followed by denaturation at 94°C for 30 s, annealing at 57 °C for 30 s, and extension at 72°C for 60s, and a final extension step at 72°C for 5 min. The amplified fragment was cut via *Dde1* restriction enzyme (Fermentas, Lithuania) at 37°C for 16 h. The wild-type allele (A allele) had one *Dde1* cleavage site and digested to 600 and 256 bp fragments, whereas the mutant allele (C allele) had two *Dde1* cleavage sites and 256 bp fragment was cleaved to 146 and 110 bp fragments, too. Digested products were separated via electrophoresis in a 2% agarose gel and visualized using safe stain staining.

## Results

Demographic and clinical characteristics of women with UL and healthy controls are presented in Table1. There was no statistically significant difference between maternal age and menarche age between the two groups. As expected, there were significant differences between the case and control groups in terms of pain and bleeding (P<0.0001). 

The frequencies for the distribution of alleles and genotypes of *ACE I/D* and *AGTR1 A1166C* polymorphisms are summarized in Table 2.

The genotypes frequencies of the *ACE I/D* and *AGTR1 A1166C *polymorphisms conformed to Hardy–Weinberg equilibrium. Also, the frequencies of II, ID, and DD genotypes were 20.8, 51, and 28.2 percent in UL women and 37, 45.5, and 17.5 in healthy women, respectively. Moreover, the risk of UL were 2 and 2.9 fold higher in ID and DD genotypes compared to II genotype, respectively (OR, 2 [95% CI, 1.3 to 3.2]; P = 0.004 and OR, 2.9 [95% CI, 1.6 to 5]; P = 0.0002). In addition, the frequency of D allele was 53.7% in women with UL and 40.3% in controls; the difference was found to be statistically significant (P = <0.0001). 

In addition, the frequencies of *AGTR1 1166*AA, AC, and CC genotypes were 86.6, 13.4, and 0 percent in UL women and 86.2, 13.3, and 0.5 in healthy women, respectively, which were not found to be statistically significant. The frequency of *AGTR1 *1166C allele did not differ between two groups, either (P=0.9)

## Discussion

Uterine leiomyomas (ULs) are the most common tumors in women, with its related etiopathogenes remaining unclear so far. Evidence showed a genetic background for UL. The incidence of various ULs confirms a genetic predisposition for UL development. In addition, the risk of UL is about 2.5 fold higher in the first-degree relatives of women with these types of tumor (Boehm et al., 1990). Therefore, several studies investigated the effects of genetic polymorphisms on UL risk with a number of studies investigating the association between the polymorphisms of various genes in RAS pathway and tumorigenesis (Deshayes and Nahmias, 2005). 

In the current study, we investigated two common polymorphisms in RAS pathway and reported their effects on tumorigenesis. The evidence showed that the *ACE* D allele increases the enzyme activity and higher activity of ACE enzyme was observed in individuals with DD genotype (Sayed-Tabatabaei et al., 2006b). In addition, it is suggested that *AGTR1 A1166C* is located in the microRNA-155 binding site and the A allele increases its affinity, leading to lower AGTR1 protein expression (Ceolotto et al., 2011).

In the current study, the frequencies of the *ACE *ID and DD genotypes were significantly higher in women with UL. Also, these genotypes were associated with the 2 and 2.9 fold increased risk of UL, respectively. However, there was no association between *AGTR1 A1166C *polymorphism and UL.

Although many studies have reported the association between *ACE I/D* and *AGTR1 A1166C* polymorphisms and various diseases (Salimi et al., 2011; Gan et al., 2015), the number of studies conducted on the association between *ACE I/D* and *AGTR1 A1166C* polymorphisms and UL are limited with inconsistent results. 

In spite of the findings of the present study, Salwa et al showed the association between AC and CC genotypes of *A1166C *polymorphism in *AGTR1* gene and the increased risk of UL. Indeed, they reported no relationship between *ACE I/D* polymorphism and this tumor (Gomaa et al., 2015). Similarly, Gültekin et al found no association between *ACE I/D* polymorphism and UL in Turkish population (Gultekin et al., 2015). Hsieh et al. reported that *ACE I*-related (II and ID) genotypes were associated with leiomyoma susceptibilities in Taiwan which is completely inconsistent with our results (Hsieh et al., 2007). In an experimental study, Isobe et al. investigated the potential role of Ang II in the proliferation of rat ELT-3 leiomyoma cells (Eker rat uterine leiomyoma-derived smooth muscle cells) in vitro and found that Ang II significantly induced ELT-3 leiomyoma cell proliferation and the expression of AGTR1 and AGTR2 mRNA and protein was confirmed. These experimental in vitro findings highlight the potential role of Ang II, through AGTR1 in the proliferation of leiomyoma cells (Isobe et al., 2007).

Several studies have investigated the effects of *ACE* and *AGTR1* polymorphisms on various tumors with inconsistent results. Kowalczyńska et al., (2011) found no association between *ACE I/D* polymorphism and the prevalence of endometriosis in polish women (Kowalczyńska et al., 2011). In another study conducted in 2014, these authors reported that *A2350G* polymorphism (G allele and AG genotype) of *ACE* gene but not *ACE I/D *and *AGTR1 A1166C* polymorphisms was associated with the development of endometriosis (Kowalczyńska et al., 2014).

In addition, other studies showed the association between *ACE I/D* and *AGTR1 A1166C* polymorphisms and various cancer risks including breast cancer (Herr et al., 2008), prostatic cancer (Uemura et al., 2006), and gastric cancer (Röcken et al., 2007). Although, our findings are not similar to those reported by Salwa et al and Hsieh et al., they are in accordance to the effect of higher ACE activity on tumorigenesis and *ACE *DD genotype on higher *ACE* activity. 

In conclusion, for the first time in Iranian women, the current study showed *ACE *ID and DD genotypes were associated with higher UL risk and that there is no relationship between *A1166C* polymorphism of *AGTR1* gene and UL. Although the mechanism by which *ACE* polymorphism may affect this complication has not been elucidated yet, the importance of the attention to *RAS* system polymorphisms in leiomyoma is considerable. Furthermore, further experimental studies may be extended to determine whether the RAS and its related gene polymorphisms also affect the leiomyoma formation.
